# Determination of the Content of Heavy Metals in Pyrite Contaminated Soil and Plants

**DOI:** 10.3390/s8095857

**Published:** 2008-09-24

**Authors:** Milan M. Antonijević, Miroslava Marić

**Affiliations:** 1 Technical Faculty, University of Belgrade, P.O.Box 50, 19210 Bor, Serbia; 2 Agricutural and Technological Research Center, 19000 Zaječar, Serbia

**Keywords:** heavy metals, pyrite contaminated soil, pollutants, amendments, oat production

## Abstract

Determination of a pyrite contaminated soil texture, content of heavy metals in the soil and soil pH, was the aim in the investigation. Acidification of damaged soil was corrected by calcium carbonate. Mineral nutrients and organic matter (NPK, dung, earthworm cast, straw and coal dust) were added to damaged soil. Afterwards, the soil was used for oat production. Determination of total heavy metal contents (Cu, Pb, Zn, Fe) in soil was performed by atomic absorption spectrofotometry. Plant material (stems, seeds) was analysed, too. Total concentration of the heavy metals in the plant material were greater than in crop obtained in unaffected soil.

## 1. Introduction

Opencast mining causes serous environmental impact such as destruction of natural soils and extraction of important volumes of materials. This causes the formation of new soils on the accumulated wastes of the mine. Elevated levels of trace elements and the acidic drainage due to oxidation of sulphide minerals are also frequently common characteristics of the most mine tailings (Michelutti et al. 1995) [1].

Copper production of industry in Bor (Serbia) during the last 100 years present a big source of environment pollution. Dust, water waste, tailing and air pollutants influence the quality of soil, water and air. Big soil area is damaged by flotation tailing from industry of Bor. The composition of the tailing was essentially pyrite (FeS2) with minor amounts other sulphides. Previous investigation of pyrite oxidation shows that different iron and sulphur species form during the decomposition of the mineral. Except that, great amounts of H+ ions release during FeS2 oxidation which lead to decrease pH of soil (Antonijevic et al. 1995) [2].

Great amounts of flotation tailing are disposed on the soil which after that remains degraded and unabled for growing plants. Lifting the dust there is air pollution so in this way soil is polluted too, as well as surface and underground waters on a wider area. Pollution of waters is also evident with releasing waste waters which contain mechanical and chemical impurities. Besides that, accidents happen. In the riverland of the Timok in east Serbia, over 2000 ha of fertile soil is damaged by the release of the flotation tailing from the Bor copper mine. In the flotation tailing, there are heavy metals which polluted the banks of that river. Because of that, it was necessary to examine the state of the polluted soil and establish what influence meliorative materials have on the amelioration of the soil with the aim of making it possible to grow plants there.

The present study has the following aims: a) to determine the particle size distribution and texture of polluted soil by flotation tailing; b) to determine pH of soil and chemical composition of soil; c) to determine influence of meliorative materials on improving soil quality; d) to determine the concentration of heavy metals in oat plants growing in polluted soil.

As meliorative supplements the following have been used: NPK, dung, earthworm cast, straw and coal dust. CaCO3 was added in all variations of the experiment for the sake of neutralisation and maintaining pH value of the soil, and mineral and organic materials have improved the chemical composition and activated the microbiological processes. On the damaged and amendment soil, oats was grown, and the content of heavy metals was examined in the plant material.

## 2. Experimental Section

### 2.1 Soil and plant analysis

A sample was taken from the polluted soil in the riverland of the river Timok which is in the eastern Serbia, nearby the copper mining and smelting combine of Bor.

Particle size distribution and soil texture were determined (Day 1965) [3].

Soil reaction was determined with a pH electrode in 2:1 water to soil extract (Sumner 1994) [4]. Exchangeable acidity was determined by the use of a 1M KCl replacing solution and titration to a phenolphtalein endpoint.

Total organic carbon (C_org_) was determined using a method of Walkley [5]. The method is based on organic matter oxidation by K-dichromate.

The ground soil sample powder were analysed for Cu, Pb, Zn, Fe, Ca concentrations using a strong acid digestion method. Approximately 1-5g of the soil samples were weighed and placed into Kjeldahl's apparatus. Concentrated nitric acid (8.0ml) and 2.0ml of concentrated perchloric acid were added. The mixtures were heated at 50°C to 190°C step by step, until they were completely dry. After the test samples were cooled, 10.0ml of 5%HNO_3_ was added and heated at 70°C for 1h with occasional mixing. Upon cooling, the mixtures were decanted, centrifuged at 3500rpm for 10min and transfered into measured flask. Total metal concentrations of the solutions were measured using inductively coupled plasma–atomic emission spectrometry (ICP–AES; Perkin-Elmer 5000).

Sulphur content was determined by oxidation sulphide to sulphate (Bugarski 1997) [6].

Available concentration of copper and zinc was determined by AAS after extracting the samples (<60μm) with a 0.05M EDTA soulution (Cottenie et al. 1979) [7].

Also, the content of heavy metals was established in the leaf and the seed of oats. Establishing of heavy metals was carried out by the method of atomic absorption of spectrophotometry (AAS). For the destruction of the samples, it was used the procedure of destruction with HNO_3_ and 30%H_2_O_2_ (Jones 1991) [8].

Available phosphorus (P_2_O_5_) and potassium (K_2_O) were photometrically determined after 0.05M acetate–fluoride extraction (Matula 1996) [9].

All sample concentrations were reported as mg/kg dry weight.

The damaged soil was examined by X-ray diffraction analysis.

### 2.2 Oat production

Oats was grown in vegetation pots which contained control soil (damaged soil without amendment) and different combinations of meliorative materials, as well as normal soil.

The variations of the experiment were as follows:

1. Control soil – damaged soil without amendment
2. Damaged soil + CaCO_3_ (40g/kg)
3. Damaged soil + CaCO_3_ (40g/kg) + NPK (300 mg/kg)
4. Damaged soil + CaCO_3_ (40g/kg) + NPK (300 mg/kg) + dung (60g/kg)
5. Damaged soil + CaCO_3_ (40g/kg) + NPK (300 mg/kg) + earthworm cast (20g/kg)
6. Damaged soil + CaCO_3_ (40g/kg) + NPK (300 mg/kg) + straw (20g/kg)
7. Damaged soil + CaCO_3_ (40g/kg) + NPK (300 mg/kg) + coal dust (60g/kg)
8. Normal Soil + NPK (300 mg/kg)

Vegetation pots, in which oats was grown, contained 10 kg of the investigated soil each and the surface of those pots was 0.21m^2^. In the soil sample (1), in the variations of the ameliorated soil (2-7), as well as in the normal soil (8) pH was measured, then – total organic carbon (TOC), the content of K, P, Ca, S and heavy metals (Cu, Pb, Zn, Fe). Based on exchangeable acidity, the amount of calcium carbonate was calculated which will be used for the neutralization of damaged soil.

Growing of oats lasted four months in the average.

## 3. Results and Discussion

### 3.1 Particle size distribution and texture

The particle size distribution (Table 1.) shows that the soil contains the low clay content in topsoil (0-44 cm). Under the topsoil, there is more clay content. The soil lacks vegetation almost totally, which exposes the very fine sand particles to hydrolytic erosion which arose by run off water.

### 3.2 Soil pH and X-ray analysis

By measuring pH, it was found out that the very acid soil – pH = 2.07 (Table 2.). X-ray analysis showed that in the damaged soil there is a sulphide mineral pyrite – FeS_2_. The increase of acidity can be explained by the present mineral of pyrite. In previous papers oxidation of pyrite has been investigated a lot, where many researchers found out that the dissolution of iron sulphide lead to the production of acid, as indicated by the following reactions (Johnson and Hallberg 2003) [10]:

$$\begin{array}{l} {\text{FeS}}_{2}+6{\text{Fe}}^{3+}+{3\text{H}}_{2}{\text{O}=7\text{Fe}}^{2+}+{\text{S}}_{2}{{\text{O}}_{3}}^{2-}+{6\text{H}}^{+}\\ {4\text{Fe}}^{2+}+{\text{O}}^{2}+{4\text{H}}^{+}=4{\text{Fe}}^{3+}+2{\text{H}}_{2}\text{O}\\ {\text{S}}_{2}{{\text{O}}_{3}}^{2-}+2{\text{O}}_{2}+{\text{H}}_{2}\text{O}={2\text{H}}^{+}+2\text{S}{{\text{O}}_{4}}^{2-}\\ {\text{Fe}}^{3+}+3{\text{H}}_{2}\text{O}=\text{Fe}{\left(\text{OH}\right)}_{3}+{3\text{H}}^{+} \end{array}$$

From the table 2 it can be seen that except the normal soil, the best buffer was the soil in which there are organic materials, because in those causes there is a minor change of pH than in soil to which organic materials weren't added. The results in the Table 2 show that during growing of oats occured the oxidation of pyrite, which was the main reason of the decrease of pH.

### 3.3. Soil chemical composition

The analysis of soil shows that the content of copper and lead is above the allowed level, which can be seen if the values are compared with the values which were established by Kloke [11], (Table 3). If the initial value of pH of the examined soil is taken into account (2.07) it can be concluded that this soil can be used for growing plants only if it has been carried out the neutralisation of the acid which became by the oxidation of the pyrite. The content of the humus in this soil is very small (0.6%) which shows that this soil must be added organic materials as well. Considering the fact that the content of phosphorus and potassium is small too, this soil must be added the nutritients which contain these minerals – inorganic NPK fertilizer. In experimental part amount of these materials are given.

### 3.4 Concentration of elements in soil during oat production

The content of the total copper in the soil in variations 1-7 differ very much from the content of the copper in normal soil (variation 8) (Table 4), both at the beginning and in the end of the experiment, and pass over the limits of maximum allowed amounts in the soil, which is 100 mg/kg. The values of the total copper in variations 2-7 at the beginning of the experiment varies from 384.30 to 427.49mg/kg, and in the end of the experiment it is 134.84 to 151.06mg/kg. The decrease of the content of the total copper in the end of the experiment compared to the beginning state varies from 59.16% to 66.93%.

The amount of zinc in the damaged soil is 8.87mg/kg (at the beginning of the experiment), that is 9.27mg/kg (at the end of the experiment). Normal soil (variation 8) contains greater quantities of the total zinc 56.15 that is 58.20mg/kg), which shows that the damaged soil is not rich with zinc, or this element was leached away from the soil, because in acid soil the mobility of zinc is increased. It is noticed that by adding CaCO_3_, NPK fertilizer and organic materials there is an increase of the total zinc, and mostly by the treatment of earthworm cast -19.46mg/kg Zn. It was found out that, compared to the amount of the total zinc, the content of available zinc is high, probably because of the acid reaction. By adding only CaCO_3_ there was a decrease of the content of the available Zn. In the end of the experiment in the variations with the organic materials, there is an increase of the content of the available zinc.

The amount of the total lead is within allowed bounds for the normal soil. However, because of the acid medium, the mobility of Pb^2+^ ions is increased. The acidity of the soil is gradually increased because of the oxidation of sulphide minerals, which leads to the gradual increase of the concentration of Pb^2+^ ions.

As a result, there is a possibility that lead is accumulated in great amount in plants.

### 3.5 Heavy metals concentration in plant

Content of copper in grains is in average smaller than in stems, which is shown by the results(Table 5.). From the results it can be seen that the accumulation of copper is biggest in the variants with the added organic material – earthworm cast and straw, whereas in the variant with the dung, accumulation of copper less.

From the Table 5. it can be seen that the content of zinc in stems of oats in variants with damaged soil varies from 64.2mg/kg (variant 3) to 98.60 mg/kg (variant 2), while in the oats grown on normal soil the concentration of zinc is 46.20mg/kg. The damaged soil, as have been stated, isn't rich with zinc, and normal soil contained bigger quantities of zinc. The presence of greater concentrations of zinc in oats grown in variants with damaged soil can be explained by the acidification of the soil, since it is known that the value of pH medium is one of the very important factors which influence the absorption of zinc. Namely, in the acid environment, the absorption of zinc is more intensive (Chaudhry and Loneragan 1972) [12].

The content of zinc in grains of oats when treating damaged soil varies from 42.05mg/kg (variant 6) to 56.50mg/kg (variant 4), while in the oats from the normal soil, the concentration of zinc is 42.30mg/kg. When added organic materials are considered, straw and coal dust influenced the smaller absorption of zinc by the plants, and in these variants, the concentrations of the absorbed zinc are similar to the concentrations of that element in the grain of oats grown on normal soil. In all the variants it was noticed that the concentration of zinc in stems was bigger than the concentration of zinc in the oats grain.

From the results shown in Table 5 it can be seen that the content of lead in oats, which was grown on the damaged soil, varies from 48mg/kg (variants 6 and 7) to 67mg/kg (variant 2) and that it can be found in the area of toxic concentration (Kabata-Pendias and Dudka 1991) [13]. The absorbtion of lead by the oats grown on the damaged soil can be explained by the acidification of the soil. It is known that on acid soil plants absorb much bigger quantities of lead (Wiklander and Vahtras 1997) [14]. Since it was found out that in variants with the damaged soil there is the decrease of pH values, and taking into account the results obtained for the content of lead at the beginning and in the end of the experiment (Table 4), it can be said that there was a bigger accumulation of lead in plants.

From the Table 5 it can also be seen that the content of iron is pretty high in oats grown on normal soil, but the content of that element is bigger in oats obtained on the pollluted soil. Compared to normal soil, the content of Fe in the examined soil is far bigger, so it is the main reason of such a balance. Besides, by acidification of soil, the mobility of iron ions is increased as a result of dissolution of iron(III) hydroxide.

## 4. Conclusions

Damaged soil has a very acid reaction (pH=2.07). By adding CaCO3 it was possible to neutralise the acidity of soil.

Addition of NPK fertilizer, earthworm casts, dung, coal dust and straw to the damaged soil has enabled the growth of oats.

During the production of oats, pH of soil decreases as a result of oxidation of the present mineral of pyrite.

The content of heavy metals (Cu, Zn, Pb) is in plant material (grains and stems) bigger than in oats, which was grown on normal soil.

## Figures and Tables

**Table 1.** Texture and particle size distribution.

Depth (cm)	Particle size(mm)				Texture
	Coarse sand >0.2	Fine sand 0.2-0.02	Silt 0.02-0.002	Clay <0.002	
0-22	2.55	69.47	21.3	6.7	Loamy sand
22-36	15.4	67.3	11.7	5.7	Loamy sand
36-44	5.2	72.2	16.0	6.6	Loamy sand
44-104	6.6	57.9	22.5	13.0	Sandy loam
104-129	21.1	42.2	19.1	17.6	Sandy loam
129-144	33.6	38.0	15.3	13.1	Sandy loam

**Table 2.** Soil pH during oat production.

	Type of soil	pH	
		Before seeding	120 days after seeding
1	Control soil – damaged soil without amendment	2.07	2.16
2	Damaged soil + CaCO_3_	6.35	3.01
3	Damaged soil + CaCO_3_ + NPK	6.55	2.90
4	Damaged soil + CaCO_3_ + NPK + dung	6.68	3.95
5	Damaged soil + CaCO_3_ + NPK + earthworm cast	6.60	3.60
6	Damaged soil + CaCO_3_ + NPK + straw	6.56	4.90
7	Damaged soil + CaCO_3_ + NPK + coal dust	6.50	4.36
8	Normal soil	6.90	6.95

**Table 3.** Chemical composition of damaged soil.

P_2_O_5_ Available mg/kg	K_2_O Available mg/kg	Ca %	Cu Total mg/kg	Cu Available mg/kg	Zn Total mg/kg	Zn Available mg/kg	Pb mg/kg	Fe %	S %
36	56	2.25	433	41.2	8.87	6.6	66.9	4.65	3.45

**Table 4.** Content of heavy metals in soil during oats production.

	Type of soil	Total Zn (mg/kg)		Total Cu (mg/kg)		Pb (mg/kg)	
		Before seeding	120 days after seeding	Before seeding	120 days after seeding	Before seeding	120 days after seeding
1	Control soil – damaged soil without amendment	8.87	9.27	433.82	428.16	66.92	42.16
2	Damaged soil + CaCO_3_	11.59	10.82	406.21	149.16	55.11	32.28
3	Damaged soil + CaCO_3_ + NPK	12.51	11.44	384.30	140.60	64.55	35.81
4	Damaged soil + CaCO_3_ + NPK + dung	12.54	12.36	410.80	150.11	69.28	34.63
5	Damaged soil + CaCO_3_ + NPK + earthworm cast	19.46	15.76	389.02	137.74	64.55	40.53
6	Damaged soil + CaCO_3_ + NPK + straw	15.29	10.20	427.49	151.06	52.74	38.17
7	Damaged soil + CaCO_3_ + NPK + coal dust	16.38	16.99	399.07	134.84	65.78	39.36
8	Normal soil	56.15	58.20	49.36	67.32	36.13	33.38

**Table 5.** Content of heavy metals in stems and grain of oat.

Type of soil*		Heavy metals (mg/kg)					
		Cu		Zn		Pb	Fe
		stems	grain	stems	grain	stems	grain
2	Damaged soil + CaCO_3_	116.96	0	98.60	0	67.00	882.4
3	Damaged soil + CaCO_3_ + NPK	157.61	52.00	70.30	50.00	66.90	1008.8
4	Damaged soil + CaCO_3_ + NPK + dung	134.85	47.50	64.20	56.50	57.40	714.8
5	Damaged soil + CaCO_3_ + NPK + earthworm cast	201.08	52.50	82.80	51.50	56.40	1293.6
6	Damaged soil + CaCO_3_ + NPK + straw	200.81	57.25	87.60	42.05	48.00	665.3
7	Damaged soil + CaCO_3_ + NPK + coal dust	172.85	45.00	91.30	43.15	48.00	636.3
8	Normal soil	22.6	18.50	46.20	42.30	12.60	489.1

* In damaged soil (variant 1) oats didn't grow, so because of that there are no results for that soil
